# Identification of Thyroid Carcinoma Related Genes with mRMR and Shortest Path Approaches

**DOI:** 10.1371/journal.pone.0094022

**Published:** 2014-04-09

**Authors:** Yaping Xu, Yue Deng, Zhenhua Ji, Haibin Liu, Yueyang Liu, Hu Peng, Jian Wu, Jingping Fan

**Affiliations:** Department of Otolaryngology head and neck surgery, Shanghai Changzheng Hospital, Second Military Medical University, Shanghai, China; Macquarie University, Australia

## Abstract

Thyroid cancer is a malignant neoplasm originated from thyroid cells. It can be classified into papillary carcinomas (PTCs) and anaplastic carcinomas (ATCs). Although ATCs are in an very aggressive status and cause more death than PTCs, their difference is poorly understood at molecular level. In this study, we focus on the transcriptome difference among PTCs, ATCs and normal tissue from a published dataset including 45 normal tissues, 49 PTCs and 11 ATCs, by applying a machine learning method, maximum relevance minimum redundancy, and identified 9 genes (*BCL2, MRPS31, ID4, RASAL2, DLG2, MY01B, ZBTB5, PRKCQ* and *PPP6C*) and 1 miscRNA (miscellaneous RNA, *LOC646736*) as important candidates involved in the progression of thyroid cancer. We further identified the protein-protein interaction (PPI) sub network from the shortest paths among the 9 genes in a PPI network constructed based on STRING database. Our results may provide insights to the molecular mechanism of the progression of thyroid cancer.

## Introduction

Thyroid tumors include encapsulated benign tumors and carcinomas, and carcinomas can be classified into papillary carcinomas (PTC) and anaplastic carcinomas (ATC). Although frequency of ATC is low (<5%), it is in a very aggressive status of thyroid carcinomas, responsible for about half of its death and its patients have a short survival time after diagnosis (6 month in average) [1]. ATC is evolved from PTC, and they are found to share genetic alterations [2]. However, limited studies reported their difference at transcriptome level [2]–[5], resulting a lack of systematic analysis of its tumor evolution.

In order to bring insight into the progression of thyroid carcinomas at systems level, we adopted a two-step computational strategy [6]. By using an effective machine learning method – mRMR (maximum relevance, minimum redundancy), we first identify genes responsible for the progressing transcriptome difference among normal tissue, PTC and ATC using the mRNA microarray data from Hebrant *et al.*'s study [5]. The machine learning method mRMR does not only identify genes with independent effect along, but also take the redundancy effect among genes selected into account. Additional to the pipeline used by Li et al. [6], we applied different validation methods, such as leave-one-out validation, 10 fold cross validation and stratified 10 fold cross validation, to determine the number of genes which separate the three tissue status, due to one validation method along may provide biased information of prediction accuracy of the machine learning model. Second, we address the function of these genes at systems level by integrating known protein-protein interaction (PPI) from STRING database. A network of shortest paths among the genes from a background PPI network could be further revealed.

## Materials and Methods

### Transcriptome Array Dataset

We adopted the gene expression data of thyroid cancer from Hebrant *et al*.'s study [5], which include the transcriptome array data of 11 anaplastic thyroid carcinomas (ATC), 49 papillary thyroid carcinomas (PTC) and 45 normal thyroids (Normal) based on Affymetrix Human Genome U133 Plus 2.0 Array. This dataset was retrieved from NCBI Gene Expression Omnibus (GEO) with an accession number GSE33630. The array platform is with 54,675 probes corresponding to 20,283 protein coding genes. The array signals were normalized with RMA using the Affymetrix Bioconductor package. For the expression value of a gene, we used the average value of normalized signals of its corresponding probes.

### STRING PPI data

The PPI data was retrieved from STRING database (version 9.0) (http://string.embl.de/) [7]. The PPI data includes both known and predicted protein interactions. We constructed a PPI network based on the STRING PPI data using a R package ‘igraph’ [8]. In the network, proteins are presented as nodes of the networks and edges corresponding to the protein-protein interactions.

### The mRMR algorithm

We used mRMR (maximum relevance minimum redundancy) method to define a gene set which can separate the three sample sets (ATC, PTC and Normal). The mRMR was first used in analyzing microarray data by Peng *et al*. [9]. Its idea is to rank features according to their relevance to the target sample variable, and meanwhile take redundancy among the features into consideration. So genes in the selected gene set has the best trade-off between maximum relevance to phenotype and minimum redundancy within genes in the selected set.

Using mutual information (MI) defined using equation (1), we quantified relevance as well as redundancy,
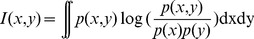
(1)where *p(x, y)* is a joint probabilistic density of vectors *x* and *y*, and *p(x)* and *p(y)* are marginal probabilistic densities.

Relevance *D* between a gene *f* and its target variable *c* is defined as,
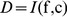
(2)


And redundancy *R* between gene *f* and genes in gene set *T* is defined as,
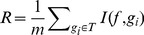
(3)where *m* is the number of genes in *T*. The trade-off between relevance and redundancy is obtained as follows,
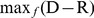
(4)


Repeating the above calculation a gene set is selected to distinguish target variables under mRMR condition with a given number *N* of genes.

Using incremental feature selection (IFS), the number *N* can be determined. Its idea is to compare prediction accuracy defined in the following selection among different *N*s, and choose the one with highest accuracy.

### Prediction of phenotypes

We used the widely used Nearest Neighbor Algorithm (NNA) to predict the target variable [10]. “Nearness” is calculated as follows,
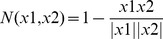
(5)where *x_1_* and *x_2_* are two vectors of genes representing two samples. The smaller *N(x1, x2)* is, the more similar the two samples are [11], [12].

### Model Validation

In Li et al. 's study [6]., leave-one-out validation was applied to validate the prediction accuracy of the study. Although the advantages of this validation method is explain in some studies [6], [13], we noticed that there are other theoretical studies demonstrated there are bias in the estimation of accuracy in the leave-one-out validation in many circumstances [14], [15]. In order to provide more information of the accuracy of the prediction model and to give an accurate estimation of the number of genes separate different tumor status, we applied two additional validation methods – 10 fold cross validation [14] and stratified 10 fold cross validation because of the stratification of tumor status (normal, PTC and ATC) [15].

### Shortest paths tracing

Genes do not function only by itself, but also by its interaction with others as well as environmental factors. Protein-protein interaction (PPI) network would bring us insights into the comprehensive biological systems. We attempted to provide such insights by searching the shortest paths which link the genes selected using mRMR and IFS in PPI network constructed according to STRING PPI data. The shortest paths were estimated using Dijkstra's algorithm [16].

### Enrichment analysis

GO (Gene Ontology) term enrichment and KEGG pathway enrichment were performed using DAVID tools [17]. We estimated the *P* values, corrected *P* values with Benjamin multiple testing correction which controlled family-wide false discovery rate, and fold enrichment values for each functional or pathway terms.

## Results

### Ten candidate genes identified by mRMR, NNA and IFS

**Figure 1:**
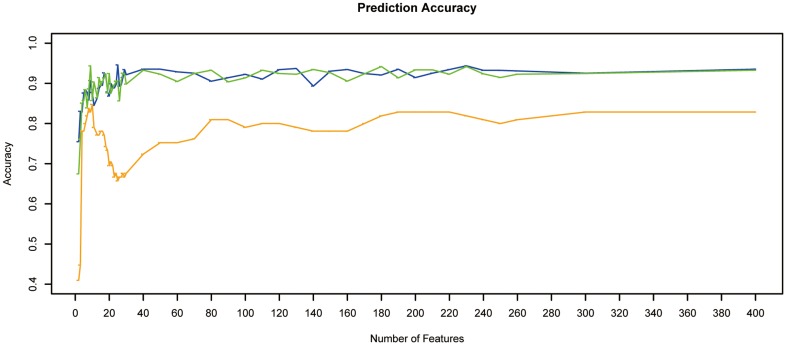
IFS curve of the classification of ATCs, PTCs and normal tissue samples. The X-axis indicate the number of genes used for classification/prediction, and Y-axis is the prediction accuracies by NNA evaluated using leave-one-out (orange), 10 fold (green) and stratified 10 fold (blue) cross validation.

On the basis of mRMR estimation, we tested the predictor of NNA described in the Materials and Methods section, with one feature, two features, … to 400 features. The result of IFS curve representing prediction accuracy estimated by leave-one-out, 10 fold and stratified 10 fod cross validation, compared with the number of features is shown in Figure 1. We noticed that although the estimation accuracies different among the three different methods, but the minimum number of genes required separating tumor status is approximately the same – about 9 or 10 (Figure 1 and Table S1). We selected 10 genes to include more candidates for further analysis and studies, and the accuracy was 0.848, 0.857 and 0.877 for leave-one-out, 10 fold and stratified 10 fold cross validation separately. The top 10 genes selected using mRMR include 9 known genes (*BCL2, MRPS31, ID4, RASAL2, DLG2, MY01B, ZBTB5, PRKCQ, PPP6C*), and a miscRNA (miscellaneous RNA, *LOC646736*) (Table 1). Interestingly, the 10 candidate genes have no overlap with the 9 differentially expression gene between ATC and PTC identified in the Hebrant *et al*.'s study. One of the possible reasons is that in our detection, we considered the variation in transcriptome differences in normal tissue, ATC and PTC together.

**Table 1:** The 10 Genes selected using mRMR and IFS.

Gene Name	Entrez Gene ID	mRMR score
BCL2	596	1.09662945
MRPS31	10240	0.222372096
ID4	3400	0.32164204
RASAL2	9462	0.390513354
DLG2	1740	0.334284222
MY01B	4430	0.354486787
ZBTB5	9925	0.384452316
LOC646736		0.339571667
PRKCQ	5588	0.359410448
PPP6C	5537	0.340892868

### Shortest path genes

**Figure 2:**
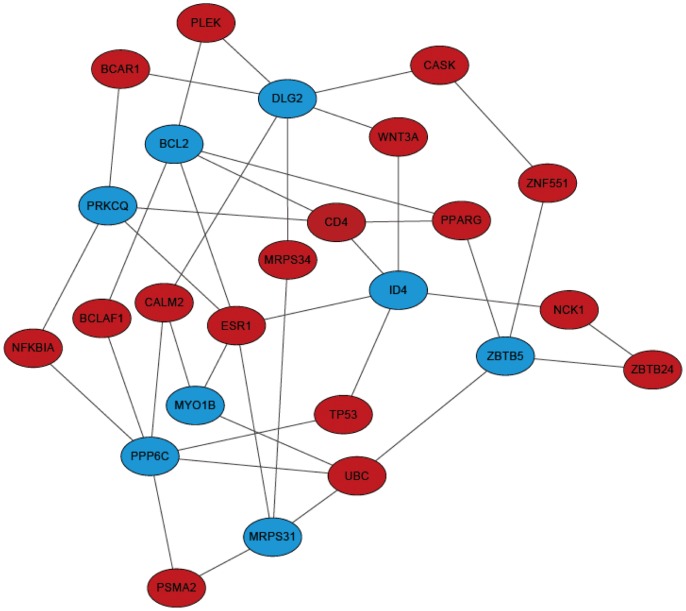
17 shortest paths genes among the 9 genes identified with mRMR methods. We identified 17 genes located on the shortest paths of STRING PPI network among the 9 mRMR identified genes. Genes in blue are those identified with mRMR methods, and genes in red are located on their shortest paths. The network is constructed based on STRING PPI data.

We constructed an undirected network based on PPI data from STRING using ‘igraph’ [8]. Then we traced shortest path between each pair of two genes from the 9 candidate genes identified using mRMR, in the PPI network using Dijkstra's algorithm [16]. There are 16 genes located on the shortest path among the 9 candidate genes, and we presented them according to their network betweenness in the shortest paths composed sub-PPI network (Table 2 and Figure 2).

**Table 2:** Proteins selected on the shortest paths among the mRMR selected proteins.

Ensembl Gene ID	Ensembl Protein ID	Associated Gene Name	betweenness
ENSG00000091831	ENSP00000206249	ESR1	5
ENSG00000010610	ENSP00000011653	CD4	4
ENSG00000150991	ENSP00000344818	UBC	4
ENSG00000143933	ENSP00000272298	CALM2	3
ENSG00000132170	ENSP00000287820	PPARG	3
ENSG00000029363	ENSP00000031135	BCLAF1	2
ENSG00000050820	ENSP00000162330	BCAR1	2
ENSG00000100906	ENSP00000216797	NFKBIA	2
ENSG00000106588	ENSP00000223321	PSMA2	2
ENSG00000112365	ENSP00000230122	ZBTB24	2
ENSG00000115956	ENSP00000234313	PLEK	2
ENSG00000141510	ENSP00000269305	TP53	2
ENSG00000204519	ENSP00000282296	ZNF551	2
ENSG00000154342	ENSP00000284523	WNT3A	2
ENSG00000158092	ENSP00000288986	NCK1	2
ENSG00000147044	ENSP00000367408	CASK	2
ENSG00000074071	ENSP00000380531	MRPS34	2

### Enrichment of the 9 candidate genes and shortest paths genes

Using DAVID tools [17], we analyzed the functional enrichment of the 9 candidate genes together with 16 shortest path genes in KEGG pathway and GO term separately. For KEGG enrichment, the 25 genes are enriched in 7 KEGG pathways listed with their *P* value and *fold enrichment* value in Table 3. Interestingly, we found most of these pathways are important pathways related with cancer, such as T cell receptor signaling pathway, apoptosis, pathways in cancer, small cell lung cancer, prostate cancer, and thyroid cancer. T Cell Receptor (TCR) activation promotes several important signals that determine cell fate through regulating cytokine production, cell survival, proliferation, and differentiation. And T cells are especially important in cell-mediated immunity, which is the defense against tumor cells. More detailed functions of TCR in cancer is reviewed in Reference [18]. Moreover, thyroid cancer pathway was also found enriched by the set of the 25 genes. For GO term enrichment, 262 GO terms are enriched (Table S2). Several of them are related with cancer progression, like GO:0042127 regulation of cell proliferation, GO:0042980 regulation of apoptosis and GO:0043067 regulation of programmed cell death. These results provide circumstantial evidence supporting our data analysis pipeline.

**Table 3:** KEGG pathway enrichment of the 25 genes selected on the shortest paths.

Term	Gene Count	*P* Value	Fold Enrichment
T cell receptor signaling pathway	4	0.002282004	13.45238095
Neurotrophin signaling pathway	4	0.003385035	11.71658986
Pathways in cancer	5	0.007626354	5.536803136
Small cell lung cancer	3	0.018690317	12.97193878
Apoptosis	3	0.019971101	12.52463054
Prostate cancer	3	0.02084525	12.24317817
Thyroid cancer	2	0.071736805	25.04926108

## Discussion

### Genes identified by mRMR and IFS

We identified 9 genes, *BCL2, MRPS31, ID4, RASAL2, DLG2, MY01B, ZBTB5, PRKCQ* and *PPP6C*, and a miscRNA *LOC646736* related with thyroid carcinoma in this study. Many of them are previously known important genes with thyroid development or cancer progression.

*BCL2*, B-cell CLL/lymphoma 2, is a protein coding gene preventing cell apoptosis, and found in many Eukaryotic species. In our mRMR results, it has the highest mRMR score (1.097), suggesting it is the most important feature to separate ATC, PTC and normal tissues. Damage to BCL2 has been identified as a cause of a number of cancers, including ovarian [19], breast [20], prostate [21], chronic lymphocytic leukemia [22]. It has also been found to be differentially expressed between PTCs and normal tissues [23], and genetic variants in BCL2 could contribute to the risk of thyroid cancer [24].

Inhibitor of DNA binding/Inhibitor of differentiation 4 (*ID4*) is a critical factor for cell proliferation and differentiation in normal vertebrate development [25]. Its protein belongs to a family of helix-loop-helix (HLH) proteins (ld1, ld2, ld3 and ld4). ID proteins contain a HLH domain enabling interaction with other basic HLH (bHLH)-proteins, and act as dominant negative inhibitors of gene transcription [26]. Family members of *ID* genes have critical row in the tumor genesis of thyroid cancer. For example, *ID1* regulates growth and differentiation in thyroid cancer cells [27], and *ID3* was also identified as an early response protein and tumor marker for thyroid carcinomas [26]. *ID4* is most recently discovered member of *ID* genes, mainly express in thyroid and several other tissues [28], and a previous study has already reported it as a marker in breast cancer [25].

### Genes identified on PPI shortest paths

*ESR1*, EStrogen Receptor 1, is the gene with the largest betweenness in the PPI network of shortest paths. It encodes estrogen receptor alpha (ERα), which mediates interaction between estrogens and its target sites together with ERβ. ERα and ERβ are both expressed in thyroid cancer cells, and the proliferation of thyroid cancer cells is promoted by an ERα agonist and reduced by enhanced expression of ERβ or by an ERβ agonist [29]. Polymorphisms in *ESR* are also involved in tumor oncogenesis in several tissues (e.g. breast, prostate, ovary and thyroid), and may alter responsiveness of the tissues to estrogens [30]–[33].

*PPARG*, peroxisome proliferator-activated receptor gamma, encodes a member of the peroxisome proliferator-activated receptor (PPAR) subfamily of nuclear receptors. It is a regulator of adipocyte differentiation, and has been found in the pathology of numerous disease. Alterations of PPARG have been discovered in a large number of thyroid cancer samples, such as PAX8/PPARG fusion oncogene in follicular thyroid carcinoma and PTCs [34]–[37], and another PPARG agonist (RS54444) in ATCs [38].

## Conclusion

In this study, we focused on transcriptome of the progression of thyroid cancer, by applying a machine learning methods to identify candidate genes separating three status of thyroid, normal, PTC and ATC. The transcriptome data includes from 11 ATCs, 49 PTCs and 45 normal tissues. We identified 9 genes (*BCL2, MRPS31, ID4, RASAL2, DLG2, MY01B, ZBTB5, PRKCQ* and *PPP6C*) and a miscRNA (*LOC646736*) related with thyroid cancer progression, additional to the genes identified previously [5]. We further revealed the PPI network of the proteins coded by these genes by estimating the shortest path of the interactions based on a background PPI network constructed based on SRING database. Our results may provide important insights to understand the mechanism of the thyroid cancer progression at transcriptome level.

## Supporting Information

Table S1 Prediction accuracy of three validation methods.
(XLSX)

Table S2 GO enrichment of the 25 genes on the shortest paths.
(TXT)
